# Analyzing the Differential Expression of Vitiligo Genes by Bioinformatics Methods

**DOI:** 10.1155/drp/6672081

**Published:** 2025-09-05

**Authors:** Quansheng Lu, Xi He, Yao Sun, Yu Lu, Guan Jiang

**Affiliations:** ^1^Department of Dermatology, Affiliated Hospital of Xuzhou Medical University, Xuzhou, Jiangsu, China; ^2^Department of Dermatology, The People's Hospital of Jiawang District of Xuzhou, Xuzhou, Jiangsu, China; ^3^Department of Dermatology, The People's Hospital of Tongshan District of Xuzhou, Xuzhou, Jiangsu, China

**Keywords:** artificial neural network, diagnosis model, FLJ21901, gene sequencing technology, MAST1, random forest analysis, vitiligo

## Abstract

**Background:** Vitiligo is a hypopigmentation skin disease that is easy to diagnose but difficult to treat. The etiology of vitiligo is unknown, which may be related to genetic and immune factors.

**Objective:** To provide potential targets for the treatment of vitiligo through identifying signature genes based on an artificial neural network (ANN) model.

**Methods:** We downloaded two publicly available datasets from GEO database and identified DEGs. We trained the random forest and ANN algorithm using training set GSE75819 to further identify new gene features and predicted the possibility of vitiligo. In addition, we further validated the performance of our model through the test set GSE53148 and verified the diagnostic value of our model with the validation set GSE53148. Finally, we used RT-qPCR to compare the expression of two genes randomly selected in this study in patients with vitiligo and healthy people.

**Results:** Two genes were randomly selected from the 30 key genes identified by ANN and validated through RT-qPCR in 6 vitiligo patients. The results showed that compared with the control group, the mRNA expression of FLJ21901 in the disease group was significantly upregulated, and the mRNA expression of MAST1 was significantly downregulated, with statistical significance.

**Conclusions:** Through the identification of characteristic genes and the construction of a neural network model, it was found that the differentially expressed genes can provide a new potential target for the treatment of vitiligo.

## 1. Introduction

Vitiligo is an autoimmune disease and also a polygenic genetic disease, which is caused by the interaction between immune cells of genetically susceptible individuals and the environment [1]. As a common pigmented skin disease, vitiligo has a negative impact on the quality of life of patients, affects people's appearance, and causes serious emotional stress [2]. The disease is characterized by leukoplakia caused by local or generalized depigmentation. It can occur in various parts of the body and is commonly seen on the back of fingers, wrists, forearms, face, neck, and around genitalia [3]. The incidence rate of vitiligo worldwide is about 2.0%, with no significant gender difference [4]. Although various strategies have been designed for the treatment of vitiligo, including hormones, local calcineurin inhibitors, vitamin D derivatives, phototherapy, and surgical techniques, there is still a lack of effective methods to ensure a complete cure [5, 6]. Therefore, it is urgent to develop effective biological methods to improve the diagnosis and prognosis of vitiligo.

In the past, as a mathematical or computational model simulating the structural information processing of synaptic connections in the brain, an artificial neural network (ANN) model has been applied to risk assessment of various diseases [7, 8]. However, there were no related studies to predict the pathogenesis of vitiligo.

In this study, RNA sequence data of vitiligo and normal skin samples were downloaded from the Gene Expression Omnibus (GEO) database to identify differentially expression genes (DEGs). MetScape gene list analysis and random forest analysis were performed. The ANN model was constructed based on database samples to calculate gene scores of vitiligo characteristic genes. Moreover, the predictive performance of the ANN model was further verified by plotting the ROC curve and the independent microarray dataset GSE75819 of vitiligo. Specifically, the microarray dataset GSE75819 was used to calculate feature gene scores to further test the accuracy of the ANN model. Finally, qPCR was used to detect the expression of FLJ21901 and MAST1, of which the first-ranked gene could provide potential targets for the treatment of vitiligo.

## 2. Materials and Methods

### 2.1. Data From GEO Database

We downloaded two independent datasets (GSE75819, GSE53148) and obtained the corresponding clinical information from the GEO database (https://www.ncbi.nlm.nih.gov/gds). The GSE75819 dataset was used as the training set (including 15 nonlesional skin and 15 lesional skin samples), and the GSE53148 dataset was adopted as the testing set, selecting 10 human specimens out of 16 samples, containing 5 vitiligo and 5 controls.  Table 1 lists the basic information of datasets downloaded from GEO.

### 2.2. Differential Expression Analysis of Training Set

In order to compare the DEGs of the vitiligo group and the normal skin group, we first analyzed the DEGs of each group by statistical test using R packages limma, pheatmap, and ggplot2 according to the gene expression matrix of the training set. Furthermore, for visualizing the DEGs in GSE75819, we plotted the heat map and volcano map by using the ggplot2 package in R. Here, the cut-off standard of DEGs is adjusted to *p* < 0.05 and |logFC| > 1.

### 2.3. Metascape Gene List Analysis of DEGs

Metascape is a tool that enables researchers to conduct batch analysis of genes and obtain gene or protein functions by using current bioinformatics analysis methods. In addition, Metascape is used for gene annotation, pathway and process enrichment analysis, and protein-protein interaction (PPI) enrichment analysis of DEGs.

### 2.4. Random Forest Analysis of DEGs

Random forest analysis can evaluate the importance of variables by integrating decision tree algorithms and also can be used to filter DEGs for finding characteristic genes related to diseases. During the training process, we first used 500 base decision trees to build a random forest model, then, we calculated the point with the smallest cross-validation error to find the optimal number of trees in the random forest. Furthermore, we ranked the importance of DEGs and selected the top 30 DEGs with the highest importance scores, which were named vitiligo characteristic genes. Finally, in order to visualize the expression of vitiligo characteristic genes, we made heat maps using the R package limma and pheatmap.

### 2.5. Gene Score Calculation of Vitiligo Signature Genes in Samples

Herein, we first inputted the expression matrix of vitiligo characteristic genes and the corresponding logFC value into R software and then compared the relative expression amount of vitiligo characteristic genes with the median expression value. If the number of upregulated genes was higher than the median expression value, the gene score was marked as 1; otherwise it was 0. If the number of downregulated gene was lower than the median value, the gene score was marked as 1; otherwise 0. Finally, the results of all signature gene scores were obtained.

### 2.6. Construction of the ANN Model

To verify the reliability and accuracy of gene scoring results, we constructed an ANN model based on 30 vitiligo characteristic genes using the R package neural net and neural Net Tools. Specifically, we set 30 nodes in the input layer and 5 nodes in the middle hidden layer, using gene score data of 30 vitiligo characteristic genes as input. Each layer's nodes can receive training feedback through variable connection strength, and the input of the next layer comes from the output of the previous layer. Compare the gene scores of samples from the vitiligo group and the normal skin group to predict which group the samples belong to. Then, we plotted ROC curve to verify the predictive accuracy of the ANN model with the pROC package in R.

### 2.7. Gene Score Calculation in the Testing Set

Firstly, we inputted the transcriptome expression matrix of the testing set and the corresponding logFC value of DEGs into R software. And then, we compared the relative expression amount of DEGs with the median expression value. If the number of upregulated genes was higher than the median expression value, it was marked as 1, otherwise it was marked as 0; if the number of downregulated genes was lower than the median expression value, it was marked as 1; otherwise, it was 0. Finally, the scores of all characteristic genes can be obtained.

### 2.8. Predictive Performance of ANN Model in Testing Set

To further validate the accuracy of the ANN model based on gene scores, 30 vitiligo characteristic genes were input, and scores were calculated for all samples in the test set. By comparing the scores of the vitiligo group and the normal skin group, they predicted which group the samples belonged to. Compared the prediction results of the ANN model with the actual grouping information to calculate the accuracy of the model. Finally, the ROC curve was plotted using the R package pROC to validate the predictive reliability of the ANN model.

### 2.9. Immune Situation Estimation and Related Tests

In this study, we used the R package “complete” with 1000 permutations and CIBERSORT (https://cibersortx.stanford.edu/) to analyze the proportion of patients with the leukocyte signature matrix core gene transcription, which has been used to infer 22 immune cell values in the vitiligo cohort. Select cases with CIBERSORT results of *p* < 0.05. To demonstrate the differences in immune cell infiltration between the two groups, we constructed violin diagram using the “vioplot” software package and used Spearman correlation analysis in R to investigate the relationship between the discovered gene indicators and the number of invading immune cells. Finally, describe the correlation obtained using the “ggplot2” software package.

### 2.10. RT-qPCR Validation of DEG mRNA Expression

Total RNA was extracted from skin lesions and nonskin tissues of 6 vitiligo patients using the RNAiso-Plus kit. Reverse transcription of RNA into cDNA using a cDNA synthesis kit. Using 1 g cDNA as the substrate, SYBR Green reagent was used for real-time quantitative PCR detection in the ABI 7300 Real Time PCR system. The primer sequences are shown in Table 2.

### 2.11. Statistical Analysis

SPSS 21.0 software was used for data analysis in this study, and the mean ± standard deviation (*x* ± *s*) was adopted for each index. Two independent sample *t*-tests were used to compare the expression of DEG mRNA in vitiligo lesions and nonlesion tissues. *p* < 0.05 indicates a statistically significant difference.

## 3. Results

### 3.1. Identification of DEGs

**Figure 1:**
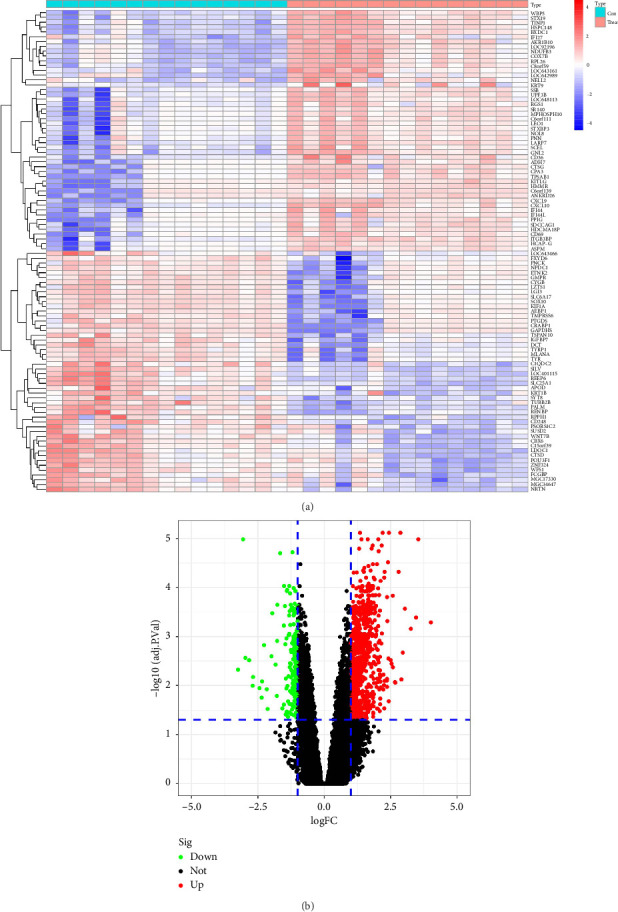
The heatmap (a) and volcano plot (b) of the DEGs. Red, upregulated DEGs; green, downregulated DEGs. Cut-off criteria: adj. *p* < 0.05 and |logFC| > 1.

Based on datasets GSE75819 and GSE53148, we identified 1071 DEGs between the vitiligo group and the normal skin group through differential gene expression analysis with the cut-off criteria of |logFC| ≥ 1 and *p* < 0.05, 224 of which were downregulated, and 847 upregulated. The heatmap in Figure 1(a) and the volcano map in Figure 1(b) show the detailed information of the DEG expression matrix.

### 3.2. Gene Annotation and Enrichment Analysis of DEGs

**Figure 2:**
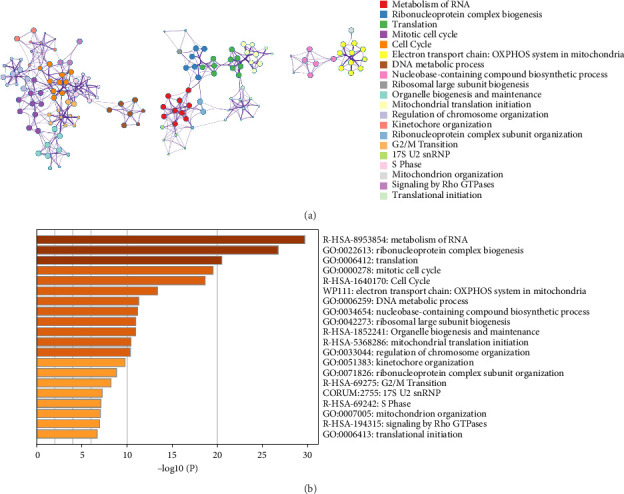
(a) An enhanced terms network. Cluster-ID is used to color the notes, and notes with the same cluster-ID are often closer to one other. (b) *p*-value-colored bar graph of enhanced phrases across DEGs lists.

**Figure 3:**
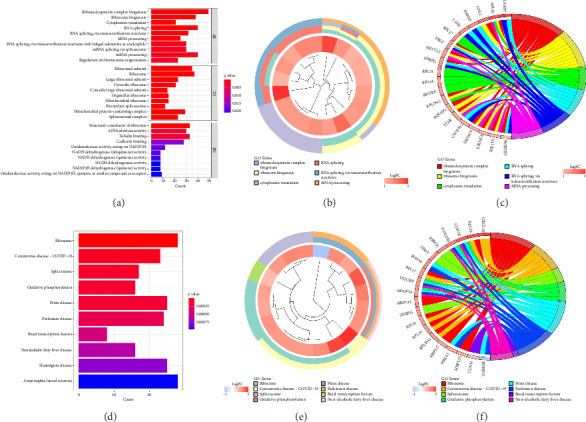
Graph depicting the findings of the enrichment analysis. (a) GO enrichment findings in a bar graph. The z-score is shown on the *x*-axis, while the log 10 (adj P) values are represented on the *y*-axis. (b) Gene clustering circle: the inner circle indicates DEGs, the red circle indicates upregulated genes, the blue circle represents downregulated genes, and the outside circle indicates GO keywords. (c) GO enrichment ring plot. The DEGs are shown on the left, with the red gene band representing upregulation and the blue gene band representing downregulation. The right-hand band, which is colored differently, represents several GO concepts. The gene's inclusion in the GO word is shown by the connecting line. (d) KEGG pathway enrichment findings in a bubble chart. The z-score is shown on the *x*-axis, while the log 10 (adj P) value is represented on the *y*-axis. A KEGG pathway is indicated by a bubble, the size of which represents the number of genes in the route. The route enrichment findings in the figure with a log 10 (adj P) > 1.3 (*p* < 0.05) are highlighted and listed in the table. (e) Gene clustering circle: the inner circle represents DEGs, the red circle indicates upregulated genes, the blue circle represents downregulated genes, and the outside circle indicates KEGG terms. (f) KEGG pathway enrichment ring plot. The DEGs are shown on the left side, with red gene bands representing upregulation and blue gene bands representing downregulation. Distinct colored bands on the right-hand side symbolize different paths. The gene's involvement in the route is shown by the connecting line.

An enhanced terms network and *P* value-colored bar graph of enhanced phrases for top 20 words from the Metascape enrichment analysis are shown in Figure 2(a) and 2(b), respectively. We conducted compression on the GO enrichment words and excluded phrases with a gene overlap over 0.75 to prevent repetition in the GO enrichment findings. Specifically, the findings of GO enrichment are shown in Figure 3.

### 3.3. Identification of Vitiligo Signature Genes via Random Forest Model

**Figure 4:**
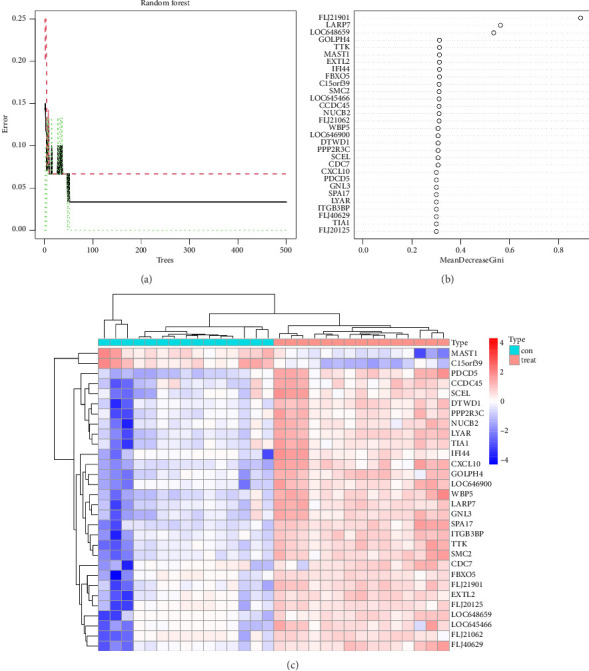
(a) The error plot of random forest trees. The abscissa represents the number of trees, and the ordinate represents cross-validation errors. The red, green and black curves represent the errors of vitiligo, normal skin and all sample groups, respectively. (b) Bubble chart of vitiligo signature genes. The abscissa represents the important score of genes, and the ordinate represents vitiligo signature genes. (c) The heatmap of Top 30 vitiligo signature genes. Red, upregulated gene; Blue, downregulated gene. Cut off criteria: adj. *p* < 0.05 and |logFC| > 1.

Based on random forest analysis, the importance of DEGs was ranked, and the Top 30 DEGs with the highest importance scores were ultimately identified as vitiligo characteristic genes. In order to visualize the error with different trees and expression of important vitiligo signature genes, we plotted the errors of random forest trees and created bubble and heatmaps of the Top 30 vitiligo characteristic genes (see Figure 4). Among them, Figure 4(a) shows the weight of the input weight layer (gene scoring matrix composed of 30 vitiligo signature genes), and the hidden layer (composed of 5 nodes), and Figure 4(b) shows the weight from the hidden layer to the output layer (representing sample grouping).  Figure 4(c) shows the heat map of the Top 30 vitiligo Signature Genes. Red is the upregulated gene; blue is the downregulated gene.

### 3.4. Construction of the ANN Model-Based 30 Vitiligo Signature Genes

**Figure 5:**
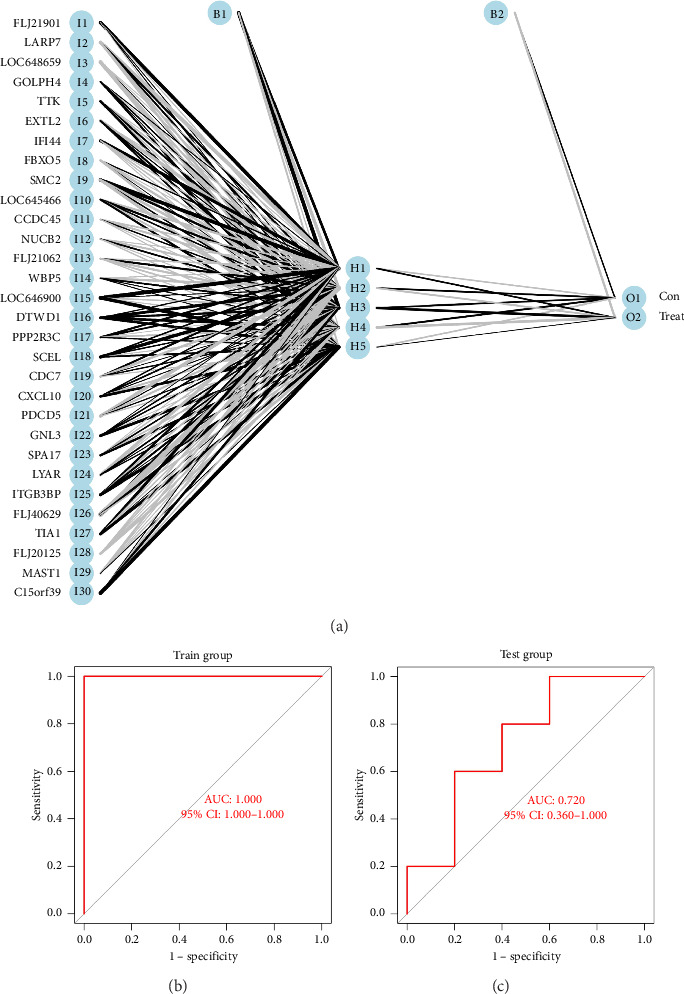
(a) ANN model based on 30 vitiligo signature genes scores. The circle in the left column is the input layer unit (gene fraction of 30 vitiligo signature genes), the circle in the middle column is the hidden layer unit (composed of 5 nodes), and the circle in the right column is the output layer unit (two groups). (b) ROC curve of ANN model. AUC: Area under curve. 95% confidence interval: 95% confidence interval. Abscissa: 1-specificity (false positive rate); Y coordinate: sensitivity (true positive rate). (c) ROC curve of ANN model in test set. 95% confidence interval: 95% confidence interval. Abscissa: 1-specificity (false positive rate); Y coordinate: sensitivity (true positive rate).

We constructed an ANN model based on the gene score matrix, which consisted of 30 vitiligo signature genes out of 30 samples using the R packages (Figure 5(a)). It can be seen in Figures 5(b) and 5(c), the areas under the ROC curve (AUC) of the training set and the test set are 1 and 0.72, respectively.

### 3.5. Immune Landscape Associated With the Characteristics of Vitiligo Patients

**Figure 6:**
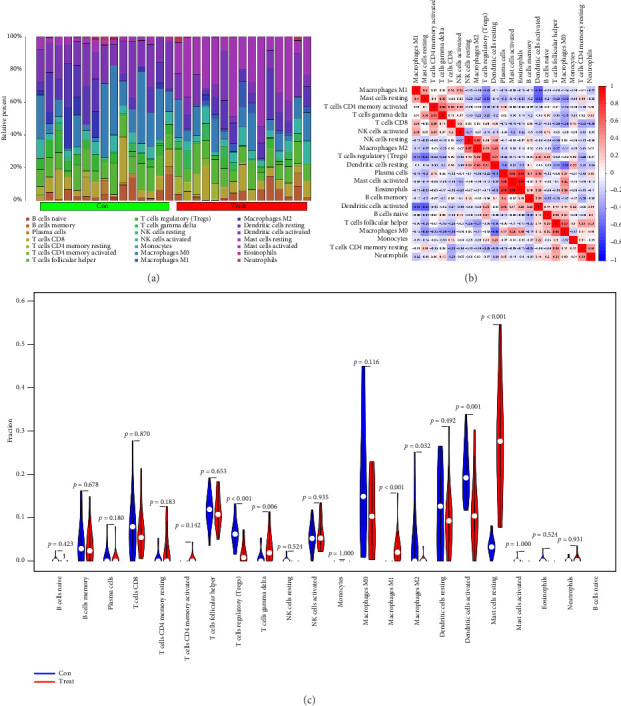
An examination of the immunological landscape of vitiligo. (a) Overview of predicted proportions of 22 immune-cell categories in con and treat groups using the CIBERSORT algorithm. (b) Correlation analysis of infiltrating immune cells. (c) Con and treat groups were compared on 22 immune-cell subtypes.

The immune-related networks of vitiligo samples were constructed by functional enrichment analysis. The CIBERSORT program was used to calculate the proportion of 22 different types of immune cells in the data. The location of 22 distinct immune cell types in vitiligo and normal skin is seen in Figure 6(a). Spearman correlation analysis was used to compare the correlation between immune cells. The largest positive correlation was between eosinophils and activated mast cells, *R* = 0.92, while the largest negative correlation was also between eosinophils and activated mast cells, *R* = −0.63 (Figure 6(b)). The proportion of T cells regulatory (Tregs) and dendritic cells activated in the vitiligo group was significantly lower than that in the healthy group (*p* < 0.001, *p*=0.001); the proportion of T cells gamma delta, macrophages M1 and mast cells resting in the vitiligo group was significantly higher than that in the healthy group (*p*=0.006, *p* < 0.001, *p* < 0.001) (Figure 6(c)).

### 3.6. RT-qPCR of Randomly Two Genes Expression Between Vitiligo Patients and Healthy People (Figure 7)

Real-time quantitative PCR was used to detect the relative mRNA expressions of differentially expressed genes FLJ21901 and MAST1 between lesion and nonlesion tissues in 6 patients with vitiligo. Compared with the skin tissue, it significantly increased the mRNA expression of vitiligo skin tissues FLJ21901, and MAST1 mRNA expression was significantly reduced.

## 4. Discussion

The pathogenesis of vitiligo is complex, and it has been found that genetic susceptibility, metabolic abnormalities, environmental changes, as well as changes in inflammation and immune responses are related to its onset [9]. In addition, the study on immunogenetics of vitiligo demonstrates that vitiligo is related to human leukocyte antigen, and the correlation varies with race, geography, age of onset of vitiligo, type of vitiligo, and severity of vitiligo [10–12]. More clinical data and experience have noted that autoimmune diseases and vitiligo are accompanied by the relationship between them [13].

This study identified 1071 DEGs between the vitiligo group and healthy group through differential gene expression analysis. Then, Metascape gene annotation, pathway and process enrichment analysis, and PPI enrichment analysis were performed on these 1071 DEGs. This study found that DEGs have important functions, key pathways, and protein interactions in vitiligo, with the immune response being more closely related to vitiligo. The direct attack of melanocytes by immune cells (such as CD8^+^ T cells) is the main cause of vitiligo. Although Figures 2 and 3 do not directly reflect the immune process, the transcriptome data indirectly reflect the downstream effects of immune attack through abnormal ribosome/RNA-related pathways, and immune assessment can screen for key driver genes. A large amount of evidence suggests that immune response plays a crucial role in the occurrence and development of vitiligo; therefore, we concluded that the biological function and pathway of these DEGs may be closely related to the occurrence of vitiligo.

Moreover, 30 vitiligo signature genes were obtained through the random forest analysis, and an ANN model was constructed based on the gene score matrix composed of 30 vitiligo signature genes. Then, we verified ANN model through the testing set. In addition, the ANN model is an effective disease prediction tool with higher accuracy and reliability than logical regression and decision tree. To our knowledge, there is currently no research on predicting vitiligo based on the neural network model. Through extensive experiments and self-validation using independent datasets and samples, the ANN model we established has predictive ability, consistent with some ANN models in tumors. However, a training AUC of 1.00 with “*n* = 30” (15 controls/15 patients) and “*p*=30 features” is a sign of overfitting. As this research is an exploratory mechanism study, the sample size will not be increased for the time being, and the subsequent research will expand the sample size based on the results of this study for further verification in order to enhance the universality and statistical power of the conclusion.

Finally, we analyzed the immune-related networks of vitiligo samples by functional enrichment analysis and randomly selected 2 of the 30 key genes obtained from the neural network for RT-qPCR validation. The immune landscape shows that the proportion of Tregs and dendritic cells activated in the vitiligo group was significantly lower than that in the healthy group; and the proportion of T cells gamma delta, macrophages M1 and mast cells resting in the vitiligo group was significantly higher than that in the healthy group. The mRNA expression of FLJ21901 was significantly upregulated in the disease group, while the expression of MAST1 was significantly downregulated. FLJ21901 was the old name of the open reading frame 141 of human chromosome 1 (C1orf141), although the function of the protein it encodes is not fully clear, some studies have found through genome-wide association studies (GWAS) that this gene locus (such as SNPS like rs1126809) is associated with the susceptibility to vitiligo, especially showing a significant association in the East Asian population [14]. And it is believed that it may be involved in the disease process by reducing the activation of Tregs or the melanocyte survival signaling pathway [15], which is consistent with our immune results. MAST1 encodes serine/threonine kinases and is involved in cytoskeleton remodeling and signal transduction. It is well known that oxidative stress is one of the causes of vitiligo, as reactive oxygen species damage melanocytes. Recent studies have shown that oxidative stress can inhibit the activity of serine/threonine kinases, leading to excessive amplification of kinase phosphorylation signals [16–18]. In this article, the MAST1 disease group is lower than the normal group, which is consistent with this. However, there is currently no direct evidence indicating that MAST1 is the causative gene for vitiligo.

In conclusion, our results has shown that the identification of characteristic genes and the construction of ANN model can provide new potential targets for clinicians to treat vitiligo patients.

## Figures and Tables

**Figure 7:**
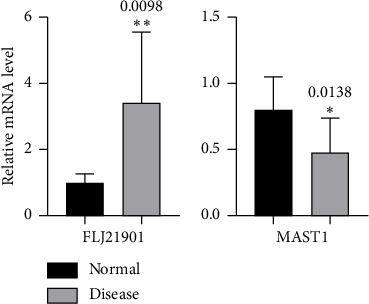
Compared with the control group, the mRNA expression of FLJ21901 was significantly upregulated in the disease group, while the expression of MAST1 was significantly downregulated, both showing significant differences.

**Table 1:** Basic information of datasets downloaded from GEO.

Dataset	Database	Area	Sample size (T/N)	No. of RNAs
Training set	GEO	USA	15/15	GSE75819
Testing set	GEO	USA	5/5	GSE53148

**Table 2:** Primer sequences in RT-qPCR.

Gene name	5′-3′	3′-5′
FLJ21901	TGACCGCTTCTGTCAACAATA	TCCTCCTAGTACCTCTGCTAAC
MAST1	CTGGAGAAGCTCCTTCAAGAC	GCAGGGCGTGAGATGATAATA

## Data Availability

The data that support the findings of this study are available from the corresponding authors upon reasonable request.
